# Spirometric Classifications of Chronic Obstructive Pulmonary Disease Severity as Predictive Markers for Clinical Outcomes: The HUNT Study

**DOI:** 10.1164/rccm.202011-4174LE

**Published:** 2021-04-15

**Authors:** Laxmi Bhatta, Linda Leivseth, Xiao-Mei Mai, Anne Hildur Henriksen, David Carslake, Yue Chen, Pablo Martinez-Camblor, Arnulf Langhammer, Ben Michael Brumpton

**Affiliations:** ^1^ Norwegian University of Science and Technology Trondheim, Norway; ^2^ Northern Norway Regional Health Authority Tromsø, Norway; ^3^ Trondheim University Hospital Trondheim, Norway; ^4^ MRC Integrative Epidemiology Unit at the University of Bristol Bristol, United Kingdom; ^5^ University of Ottawa Ottawa, Ontario, Canada; ^6^ Geisel School of Medicine at Dartmouth Hanover, New Hampshire; ^7^ Norwegian University of Science and Technology, Levanger, Norway and; ^8^ Nord-Trøndelag Hospital Trust, Levanger, Norway

The classification of chronic obstructive pulmonary disease (COPD) severity is important in guiding therapy and prognosis (1). The Global Initiative for Chronic Obstructive Lung Disease (GOLD) has recommended GOLD grades (1) based on post-bronchodilator percentage-predicted FEV_1_ (ppFEV_1_), which is widely used in respiratory medicine. However, ppFEV_1_ has been criticized because of its susceptibility to physiological variation (2–4). Studies have recommended alternative expressions of FEV_1_ that could be used for the classification of COPD severity (2, 3, 5–9). For the first time, we have compared the predictive abilities of a broad range of FEV_1_ expressions for cause-specific mortality and hospitalization.

Some of the results of these studies have been previously reported in the form of a preprint (https://doi.org/10.1101/2020.11.03.20221432).

## Methods

This study included people aged ≥40 years who participated in the HUNT2 Study (Trøndelag Health Study 2) during 1995–1997 (*n* = 44,384; 75.2% participation). A 5% random sample (*n* = 2,300) and people reporting asthma-related symptoms, diagnosis, or use of medication (*n* = 7,123) were invited to perform spirometry (10). For the analysis, we included 890 people with COPD who met both the fixed ratio (post-bronchodilator FEV_1_/FVC <0.70) and lower limit of normal criteria and had respiratory symptoms (daily cough in periods, cough with phlegm, wheezing, or dyspnea) and/or self-reported doctor-diagnosed COPD (1, 11).

Post-bronchodilator spirometry was performed 30 minutes after inhalation of 1 mg of terbutaline according to the 1994 American Thoracic Society guidelines (12, 13). Quality assurance of spirometric measurements is described in detail elsewhere (13, 14).

We defined expressions of FEV_1_ such as ppFEV_1_, FEV_1_ *z*-score, FEV_1__ _⋅_ _Ht^−2^, FEV_1 _⋅_ _Ht^−3^, and FEV_1_Q (described in detail in Reference 15) as suggested by the previous studies (1–3, 5, 6, 8, 9, 16). The Global Lung Function Initiative 2012 reference equation was used to calculate ppFEV_1_, ppFVC, FEV_1_ *z*-scores, and FVC *z*-scores (11, 13). FEV_1_ was standardized by the square of height in meters to calculate FEV_1 _⋅_ _Ht^−2^ (6, 9) and by the cube of height in meters to calculate FEV_1 _⋅_ _Ht^−3^ (5, 8). FEV_1_ was standardized by sex-specific lowest percentile (0.5 L for men and 0.4 L for women) of FEV_1_ distribution among patients to calculate FEV_1_Q, as suggested by Miller and Pederson in a large European population consisting of three cohorts (5).

### Follow-up and outcomes

The study outcomes were all-cause mortality, respiratory mortality, cardiovascular mortality, the first unplanned COPD hospitalization, and the first unplanned pneumonia hospitalization. Participants were followed for 5 years, and right-censoring events were emigration (*n* = 3) or end of follow-up. Cause-specific mortality and hospitalizations were identified using International Classification of Diseases codes from medical records and are described in detail elsewhere (15).

### Statistical analysis

Cumulative incidence curves for all-cause mortality were constructed through Kaplan-Meier estimates, and log-rank tests were used to test differences.

A regression tree method (17) that accounts for time and multiple outcomes was applied to obtain optimal cutoffs of FEV_1_Q (2.8, 4.1, and 5.2), termed FEV_1_Q grades.

We applied incident/dynamic time-dependent areas under the receiver operating characteristic curves (AUCs) that account for time to compare the predictive abilities of FEV_1_ expressions and their respective methods of classification of COPD severity to predict clinical outcomes (18–21). For cause-specific mortality and hospitalization, AUCs accounting for competing risks were calculated (20). We used crude models to compare AUCs because the clinical decision does not explicitly take into account other factors (5). We used 10,000 bootstrap iterations to calculate the 95% confidence interval for AUCs (22). A general bootstrap algorithm (23) was applied to compare the AUCs.

Statistical analysis was performed using R 3.6.1 software (http://www.r-project.org).

### Ethics

Ethical approval was obtained from the Regional Committees for Medical and Health Research Ethics (2015/1461/REK midt). All participants gave informed written consent.

## Results

**Figure 1.**
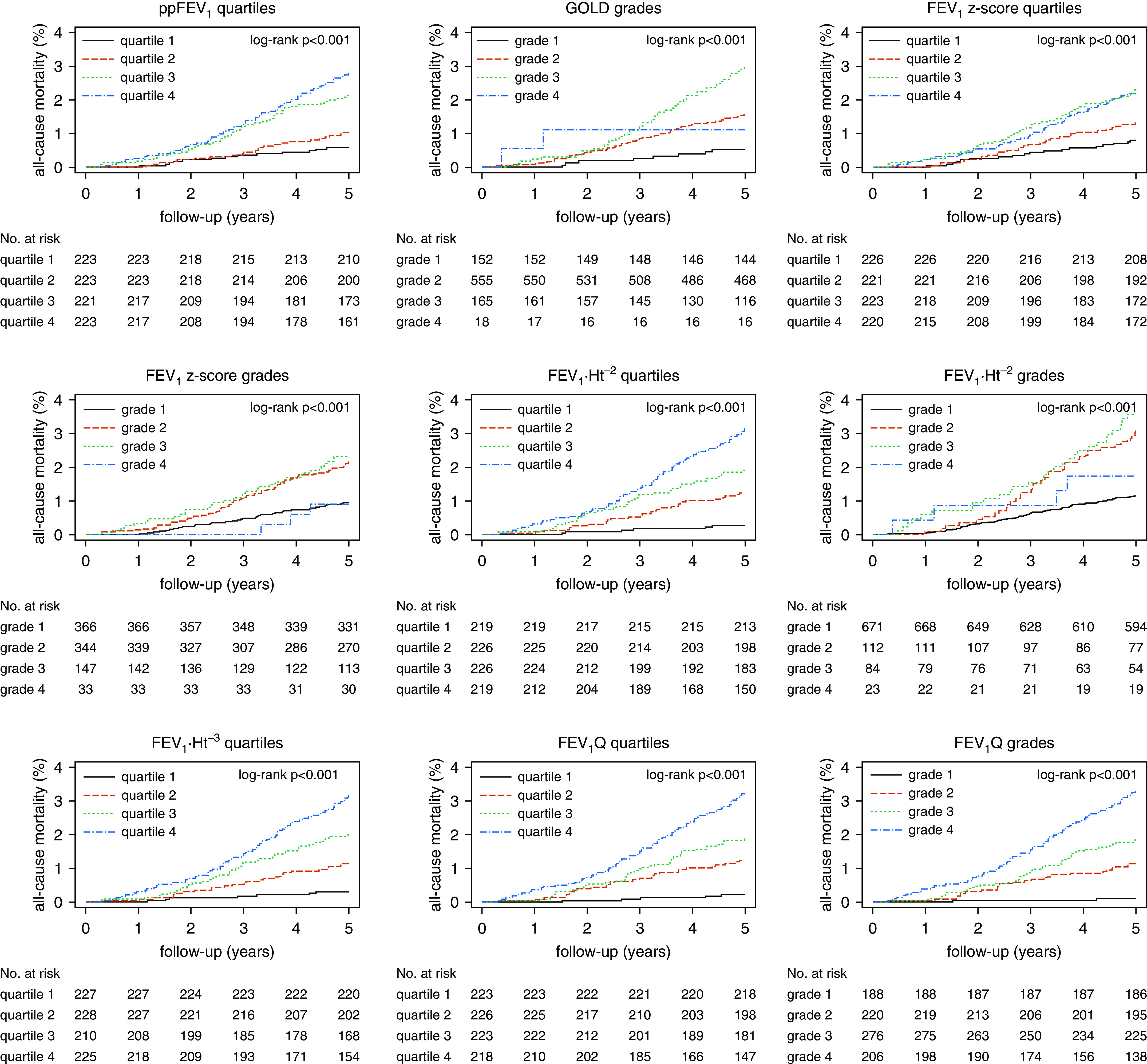
Cumulative incidence curves of classifications of chronic obstructive pulmonary disease (COPD) severity for all-cause mortality among participants with COPD aged ≥40 years in the HUNT2 Study (Trøndelag Health Study 2) (1995–1997) followed for 5 years. FEV_1 _⋅_ _Ht^−2^ = FEV_1_ standardized by square of height in meters; FEV_1 _⋅_ _Ht^−3^ = FEV_1_ standardized by cube of height in meters; FEV_1_Q = FEV_1_ standardized by sex-specific lowest percentile (0.5 L for men and 0.4 L for women) of FEV_1_ distribution; FEV_1_ *z*-score = FEV_1_z-score based on the Global Lung Function Initiative 2012 equation; GOLD = Global Initiative for Chronic Obstructive Lung Disease; ppFEV_1_ = percentage-predicted FEV_1_ based on the Global Lung Function Initiative 2012 equation.

During the follow-up period, 146, 30, and 56 subjects died because of all causes, respiratory diseases, and cardiovascular diseases, respectively, and 172 and 96 were hospitalized because of COPD and pneumonia, respectively. At baseline, the average age of participants was 63.8 years, 6 of 10 participants were men, and more than half (53.3%) of participants were current smokers (15). A trend for increasing cumulative incidence of all-cause mortality with worsening categories of classifications of COPD severity was observed (Figure 1).

**Figure 2.**
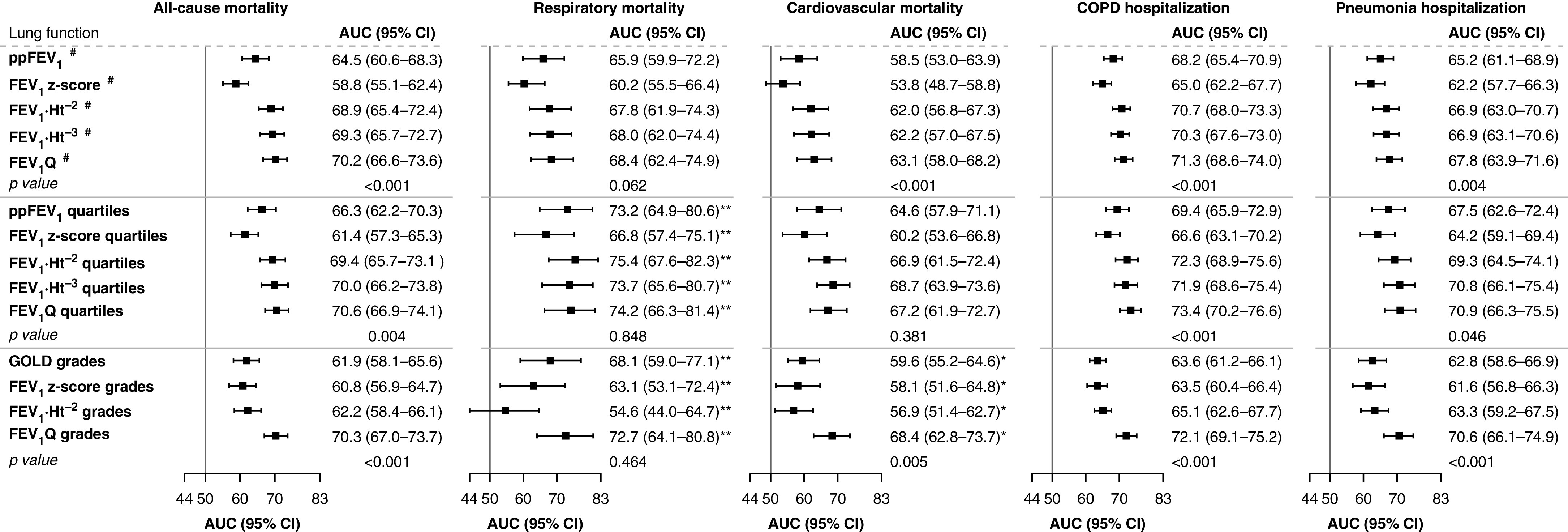
The areas under the receiver operating characteristic curves (AUCs) for different expressions of FEV_1_ and their respective methods of classification of chronic obstructive pulmonary disease (COPD) severity for all-cause mortality (*n* = 146), respiratory mortality (*n* = 30), cardiovascular mortality (*n* = 56), COPD hospitalization (*n* = 172), and pneumonia hospitalization (*n* = 96) among participants with COPD aged ≥40 years in the HUNT2 Study (Trøndelag Health Study 2) (1995–1997) followed for 5 years. ^**#**^Continuous variables. *Grades/quartiles 3–4 were combined because of zero cases in grade/quartile 4 in Global Initiative for Chronic Obstructive Lung Disease (GOLD) grades, FEV_1_ *z*-score grades, and FEV_1_ standardized by square of height in meters (FEV_1 _⋅_ _Ht^−2^) grades. **Grades/quartiles 2–4 were analyzed because of zero cases in grade/quartile 1 of GOLD grades, FEV_1 _⋅_ _Ht^−2^ quartiles, FEV_1 _⋅_ _Ht^−3^ quartiles, FEV_1_ standardized by sex-specific lowest percentile (0.5 L for men and 0.4 L for women) of FEV_1_ distribution (FEV_1_Q) quartiles, and FEV_1_Q grades. Similar differences in AUCs were observed when grade/quartiles 1–2 were combined for respiratory mortality. *P* value represents the differences between ppFEV_1_ vs. FEV_1_Q, ppFEV_1_ quartiles versus FEV_1_Q quartiles, and GOLD grades versus FEV_1_Q grades. CI = confidence interval; FEV_1 _⋅_ _Ht^−3^ = FEV_1_ standardized by cube of height in meters; FEV_1_ *z*-score = FEV_1_ *z*-score based on the Global Lung Function Initiative 2012 equation; ppFEV_1_ = percentage-predicted FEV_1_ based on the Global Lung Function Initiative 2012 equation.

When using FEV_1_ expressions as continuous measures, the AUCs for all-cause mortality were 64.5 for ppFEV_1_, 58.8 for FEV_1_ *z*-score, 68.9 for FEV_1 _⋅_ _Ht^−2^, 69.3 for FEV_1 _⋅_ _Ht^−3^, and 70.2 for FEV_1_Q (*P* value for AUCs between ppFEV_1_ and FEV_1_Q <0.001). Similar patterns of AUCs were observed for cause-specific mortality and hospitalization, except for respiratory mortality (*P* = 0.062) (Figure 2).

The FEV_1_Q grades had higher AUCs compared with GOLD grades for predicting all-cause mortality (*P* < 0.001), cardiovascular mortality (*P* = 0.005), COPD hospitalization (*P* < 0.001), and pneumonia hospitalization (*P* < 0.001) but not for respiratory mortality (*P* = 0.464) (Figure 2). Similar patterns of AUCs were observed when using FEV_1_ expressions as ppFEV_1_ quartiles and FEV_1_Q quartiles, except for respiratory mortality (*P* = 0.848) and cardiovascular mortality (*P* = 0.381) (Figure 2).

## Discussion

In this population-based study, we found that among all FEV_1_ expressions, FEV_1_Q was the best predictor of clinical outcomes studied, followed by FEV_1 _⋅_ _Ht^−2^ or FEV_1 _⋅_ _Ht^−3^, across 5 years of follow-up. For respiratory mortality, the smaller sample size gives imprecise estimates, resulting in a marginally similar predictive ability for FEV_1_Q and ppFEV_1_.

FEV_1_ is a continuous variable, the expression of FEV_1_ is used for indicating lung function impairments in respiratory medicine, and ppFEV_1_ is most commonly used for this purpose (1). The GOLD grades based on ppFEV_1_ have been widely used for clinical purposes in classifying COPD severity (1). However, they have been criticized because of their susceptibility to physiological variation and poor prediction ability (2–4, 6). The FEV_1_ *z*-score avoids this bias due to physiological variation (2, 3). In addition, ppFEV_1_ and FEV_1_ *z*-scores are based on reference values and depend on the choice of reference equation; therefore, performance might vary with reference values (11, 13, 24, 25). Miller and colleagues (5–7) found that FEV_1_ expressions such as FEV_1 _⋅_ _Ht^−2^, FEV_1 _⋅_ _Ht^−3^, and FEV_1_Q, which are independent of reference equations, were better correlated with mortality than those that are dependent on reference equations. In addition, Miller and Pedersen (5) found that FEV_1_Q predicted mortality better than other FEV_1_ expressions. Extending this knowledge, our study supports FEV_1_Q as a stronger predictor than other FEV_1_ expressions in predicting multiple clinical outcomes. This indicates that the severity in people with COPD appears to be better related to how far the FEV_1_ of that person is from the “bottom line” rather than how far it is from a “predicted value.”

The predictive ability of a classification of COPD severity based on a FEV_1_ expression largely depends on the choice of cutoffs. For example, the GOLD grades and ppFEV_1_ quartiles had different predictive abilities in our study. Huang and colleagues (4) observed similar results. Therefore, the optimal cutoffs of FEV_1_ expressions for classification of COPD severity were investigated in this study, and we found that cutoffs for FEV_1_Q (2.8, 4.1, and 5.2; FEV_1_Q grades) were generally best in predicting clinical outcomes. The optimal cutoffs should be further investigated in a large multiethnic population with a wide age range. In a clinical setting, information such as age, sex, and height of patients with COPD is easily available. Therefore, using FEV_1_Q (or other expressions of FEV_1_ that are independent of reference equations) for risk classification of patients with COPD might be easy to apply and avoid variation due to dependence on reference equations (5). Furthermore, multidimensional prognostic indices that combine reference independent FEV_1_ expressions with symptoms, exacerbations, risk factors, and/or biomarkers should be investigated further.

This study also had certain limitations. Our methods may not capture nonlinear associations between FEV_1_ expressions and mortality (26) or hospitalization, and further studies investigating these approaches are needed.

In summary, these findings highlight improved prediction of outcomes by the use of FEV_1_Q.

## Supplementary Material

Supplements

Author disclosures
