# Supplementary material for: Spirometric Classifications of Chronic Obstructive Pulmonary Disease Severity as Predictive Markers for Clinical Outcomes: The HUNT Study
Source: Am J Respir Crit Care Med. 2021 Apr 15;203(8):1033–7. doi: 10.1164/rccm.202011-4174LE (PMC8048755; doi:10.1164/rccm.202011-4174LE)
Supplement: Supplements [file rccm.202011-4174LE.html]

Spirometric Classifications of Chronic Obstructive Pulmonary Disease Severity as Predictive Markers for Clinical Outcomes: The HUNT Study | American Journal of Respiratory and Critical Care Medicine

- disclosures.pdf (230 KB)
